# Calcium homeostasis in red blood cells of dialysis patients in dependence of erythropoietin treatment

**DOI:** 10.3389/fphys.2014.00016

**Published:** 2014-01-27

**Authors:** Jue Wang, Kai van Bentum, Urban Sester, Lars Kaestner

**Affiliations:** ^1^Research Centre for Molecular Imaging and Screening, School of Medicine, Institute for Molecular Cell Biology, Saarland UniversityHomburg/Saar, Germany; ^2^Ambulatory Health Care Center SaarpfalzHomburg/Saar, Germany; ^3^Internal Medicine IV, School of Medicine, Saarland UniversityHomburg/Saar, Germany

**Keywords:** erythrocyte, end-stage renal disease, calcium, EPO, haemodialysis, thrombotic events

**Figure 1:**
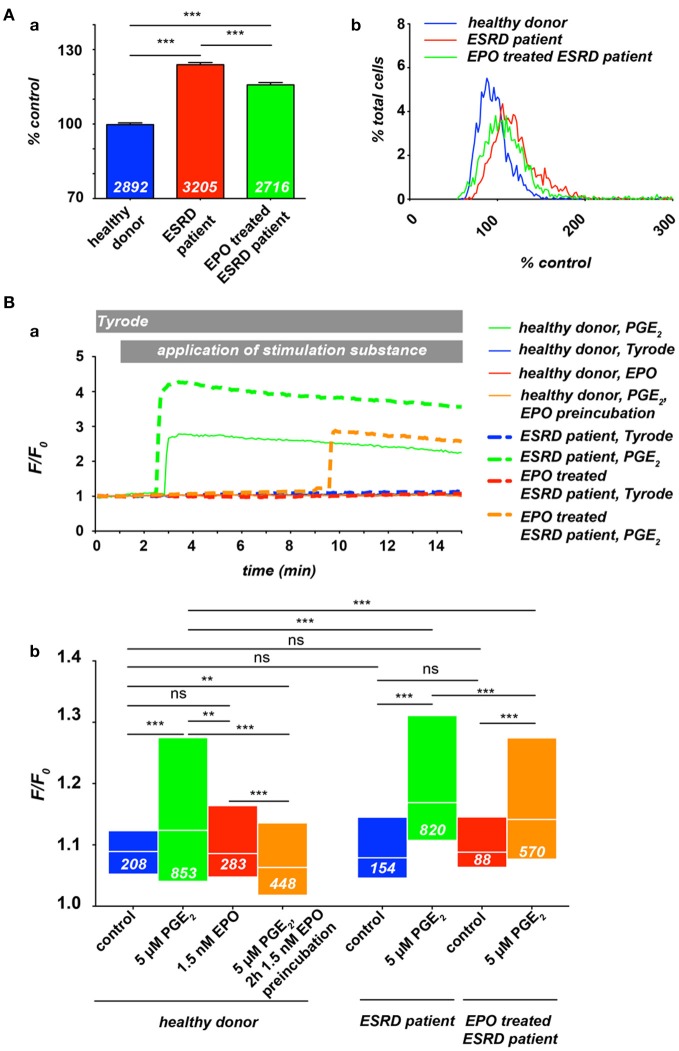
**Ca^2+^ in RBCs of healthy donors and end-stage renal disease (ESRD) patients with a renal anaemia**. If patients were under EPO treatment, the last EPO administration was within 5 days of blood sampling. All experimental procedures are previously described in detail (Wang et al., 2013). **(A)** Resting Ca^2+^ in terms of relative Fluo-4 fluorescence intensity of RBCs suspended in plasma. **(Aa)** Statistical analysis of RBC Ca^2+^ of at least 3 individuals in each group. The white numbers indicate the number of cells analyzed and the error bars represent standard error of mean. **(Ab)** Histograms of the relative fluorescence intensity distribution of the measurements presented in **(Aa)**. **(B)** Intracellular Ca^2+^-traces in RBCs under different conditions: control (Tyrode solution containing in mM: 135 NaCl, 5.4 KCl, 10 glucose, 1 MgCl_2_, 1.8 CaCl_2_ and 10 HEPES; the pH was adjusted to 7.35 using NaOH) or stimulation with 5 μM PGE_2_ and/or 1.5 nM EPO. **(Ba)** Typical example traces of Fluo-4 fluorescence intensity over time for all conditions tested. Traces were chosen due to their typical intensity of response, which are analyzed in **(Bb)**. Differences in the onset of the response were equally distributed over a wide range for all conditions tested (data not shown). However, it is worthwhile to mention that in case of stimulation only a limited number of cells are responders (Kaestner et al., 2004), which explains the difference in normalized fluorescence intensity (F/F_o_) between the example traces and the statistical analysis of the entire cell population measured. The white numbers indicate the number of cells analyzed. For the statistical evaluation a Mann–Whitney test was performed and the following convention was used: ^***^p < 0.001, ^**^p < 0.01, and ns *p* > 0.05.

Previous studies provided evidence for a massively increased intracellular Ca^2+^ concentration in red blood cells (RBCs) of patients with end-stage renal disease (ESRD) (Paschen et al., 1971; Gafter et al., 1989), whereas the dialysis procedure itself led in average to an even slightly decreased RBC's Ca^2+^ content (Paschen et al., 1971; Długaszek et al., 2008). Based on a single cell approach we could qualitatively confirm these results (Figure 1A, 2 leftmost columns), although the extend of the Ca^2+^ increase was smaller compared to the cited investigations, which is presumably caused by differences in the methodology.

There is a good knowledge of Ca^2+^ related processes in RBCs (Bogdanova et al., 2013). Additionally, an increased intracellular free Ca^2+^ concentration in RBCs has been proposed as a trigger for intracellular aggregation (Andrews and Low, 1999; Kaestner and Bernhardt, 2002) as well as for endothelium-RBC adhesion (Hebbel et al., 1980; Mohandas and Evans, 1985) and experimental evidence has been provided (Noh et al., 2010; Steffen et al., 2011; Borst et al., 2012; Kaestner et al., 2012).

The molecular identity of Ca^2+^- and non-selective cation channels in the RBC membrane is steadily increasing (Kaestner, 2011) and comprise of, e.g., the Ca_V_2.1 (Andrews et al., 2002), the TRPC6 (Foller et al., 2008), the NMDA-receptor (Makhro et al., 2013), and the Piezo1 (Zarychanski et al., 2012).

The effect of erythropoietin (EPO) on RBCs ion homeostasis is controversially discussed. The group of Florian Lang found an inhibition of non-selective cation channels by EPO with a decreased number of eryptotic RBCs if patients were treated with EPO (Myssina et al., 2003). However, once Ca^2+^ entered the RBC, EPO has no beneficial effect toward the eryptotic symptoms caused by Ca^2+^ (Vota et al., 2013). In contrast to RBCs, hematopoietic progenitor cells display an increased cation-channel activity upon EPO exposure (Cheung et al., 1997; Tong et al., 2008). Because of its hematopoiesis stimulating properties, EPO became a widely used medication for treatment of anemic patients, including chronic renal disease, hematologic disorders, and acquired immune deficiency syndrome (Palmer et al., 2010; Goodnough and Shander, 2013). However, several studies highlighted the problem of an increased risk of thrombus formation, especially venous thromboembolism, in patients undergoing EPO therapy (Singbartl, 1994; Kliger et al., 2012; Goodnough and Shander, 2013).

Therefore we investigated the free Ca^2+^ concentration in RBCs from ESRD (dialysis) patients under EPO treatment. Blood samples from healthy donors, ESRD patients and EPO treated ESRD patients were analyzed by fluorescence live cell imaging as previously described (Wang et al., 2013) (Figure 1). As depicted in Figure 1A, at rest, RBCs from ESRD patients show higher Ca^2+^ concentration compared with healthy donors, while EPO treatment let to a slightly decreased free internal Ca^2+^ concentration, indicating an inhibition of constitutively active channels in resting RBCs. Although the histograms (Figure 1Ab) give an impression of the distribution, the method lacks quantitative information concerning the Ca^2+^ concentration (Kaestner et al., 2006). However, when compared to control conditions, the width of the distribution of Ca^2+^ content is wider in ESRD patients or EPO-treated ESRD patients, leading to the conclusion that the cellular heterogeneity is greater in patients than in healthy subjects. In a further step we investigated the Ca^2+^ influx in RBC from healthy donors and ESRD patients after hormonal stimulation in dependence of EPO treatment (Figure 1B). As a stimulation substance we selected prostaglandin E_2_ (PGE_2_), which is released from activated platelets (Smith et al., 1973) but can also be released from RBCs themselves when they pass small capillaries (Oonishi et al., 1998). The curves in Figure 1Ba present typical example traces for Ca^2+^ curves in RBCs, while Figure 1Bb shows the statistical analysis. Healthy patients show an increase in Ca^2+^ after PGE_2_ stimulation as we have previously shown (Kaestner et al., 2004). EPO treatment prevents Ca^2+^ entry resulting in Ca^2+^ levels below control conditions confirming the results of the Lang group (Myssina et al., 2003). However, pretreatment with EPO even suppressed the Ca^2+^ entry provoked by PGE_2_. In ESRD patients, PGE_2_ stimulation leads to a Ca^2+^ increase, which is significantly higher than in RBCs of healthy donors. In EPO treated ESRD patients the PGE_2_ induced Ca^2+^ increase was significantly suppressed compared to RBCs of non-treated patients even in the putative absence of EPO during the experiment.

The results of Figure 1 suggest that the clinically observed thrombotic complications in patients treated with EPO seem not to be primarily caused by an elevated Ca^2+^ content of RBCs of these patients. However, it is hypothesized that EPO causes thrombosis as a result of inflammation (Tobu et al., 2004). The elevated basal Ca^2+^ level in RBCs of ESRD patients (Paschen et al., 1971; Gafter et al., 1989; Figure 1A) may enhance a blood clotting once initiated by an inflammation processes. It is a substantial finding that the response of RBC to hormonal stimulation or other treatments, like the dialysis itself, in terms of Ca^2+^ entry shows a wide variation between RBC within a population, but also between different individuals (Paschen et al., 1971; Wang et al., 2013; Figure 1Ba).

In light of the above statements concerning the thrombotic events and the molecular players, the perspective of medical treatment must be based on a personalized diagnosis followed by a personalized medication. This applies to ESRD patients as well as for treatments of other anemias. Techniques and procedures allowing such an individualized approach presumably based on the combination of RBC population measurements and single cell techniques (Minetti et al., 2013) need urgently to be developed.
